# Electrocardiographic characteristics of cirrhotic patients and their association with Child‐Pugh score

**DOI:** 10.1002/clc.24089

**Published:** 2023-07-12

**Authors:** Soodeh Jahangiri, Alireza Abdiardekani, Saideh Jamshidi, Amir Askarinejad, SeyedArad Mosalamiaghili, Mehdi Bazrafshan, Mohamadreza Karimi, Hanieh Bazrafshan, Hamed Bazrafshan drissi

**Affiliations:** ^1^ Endocrine Research Center, Institute of Endocrinology and Metabolism Iran University of Medical Sciences Tehran Iran; ^2^ Cardiovascular Research Center Shiraz University of Medical Sciences Shiraz Iran; ^3^ Department of Cardiology, Abu‐Ali Sina Charity Hospital Shiraz University of Medical Sciences Shiraz Iran; ^4^ Rajaie Cardiovascular Medical and Research Center Iran University of Medical Sciences Tehran Iran; ^5^ Department of Neurology, Clinical Neurology Research Center Shiraz University of Medical Sciences Shiraz Iran

**Keywords:** cardiac dysfunction, Child‐Pugh score, cirrhosis, electrocardiography

## Abstract

**Background:**

Cardiac dysfunction is a serious complication of cirrhosis which is usually asymptomatic. We investigated the clinical and electrocardiographic (ECG)‐related factors among patients with cirrhosis and our aim was to find any associations between ECG changes and the etiology of cirrhosis, as well as Child‐Pugh score.

**Hypothesis:**

We hypothesized that some ECG‐related factors, particularly prolonged QT interval, are more common in patients with cirrhosis. Also, these factors are associated with the severity of cirrhosis, measured by the Child‐Pugh score.

**Methods:**

From April 2019 to December 2022, we reviewed admitted patients to Namazi and Abu‐Ali Sina hospitals, Shiraz, Iran. Patients with confirmed diagnosis of cirrhosis and without concurrent disorders affecting the cardiovascular system were selected. Clinical and ECG‐related data were then extracted for participants, and Child‐Pugh score was calculated.

**Results:**

A total of 425 patients were included; the median age was 36 years, and 245 patients (57.6%) were men. Cryptogenic and primary sclerosing cholangitis were the most common etiologies. Prolonged QT followed by early transitional zone were the most common ECG changes (24.7% and 19.8%, respectively), which were significantly associated with the etiology of cirrhosis and Child‐Pugh class.

**Conclusions:**

Prolonged QT interval and presence of early transitional zone in patients with cirrhosis may indicate cardiac dysfunction, necessitating further evaluations.

## Graphical abstract

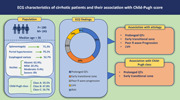

## 1 BACKGROUND

About 112 million individuals have cirrhosis worldwide, and it is the leading cause of death (2.4% of global deaths in 2019).^1^ Vasodilation and hemodynamic changes in cirrhosis cause multiorgan involvement, such as portal hypertension, pulmonary edema, esophageal varices, ascites, spontaneous bacterial peritonitis, and cirrhotic cardiomyopathy.^2^

Cirrhotic cardiomyopathy is a state of high cardiac output, low systemic vascular resistance, and impaired cardiac response to pathophysiological stress without prior heart disease. It is an underdiagnosed condition, remaining asymptomatic for extended periods.^3^ In 2019, Cirrhotic Cardiomyopathy Consortium redefined the diagnostic criteria, but these criteria are majorly based on advanced techniques such as echocardiographic strain analysis and tissue Doppler imaging.^4^ Electrocardiographic (ECG) changes are among the possible indicators of this condition, and the recent guidelines of cirrhotic cardiomyopathy support further investigational research on them as valuable noninvasive markers.^4^ Thus, we aimed to study ECG changes in cirrhotic individuals and investigated their relationship with cirrhosis etiology as well as the Child‐Pugh score.

## 2 METHODS

### 2.1 Study design and participants

The cardiovascular research group of Shiraz University of Medical Science at Namazi and Abu‐Ali Sina Hospitals, carried out this cross‐sectional study. From April 2019 to December 2022, we recruited data from admitted patients with cirrhosis. Men <45 and women <55 years old with a confirmed diagnosis of liver cirrhosis were included. Exclusion criteria were: history of concurrent cardiovascular or other disorders affecting ECG; history of electrolyte imbalance (i.e., Magnesium, Potassium, and Calcium); taking drugs that affect the ECG (e.g., beta‐blockers) and electrolytes (e.g., Furosemide and spironolactone). The institute's ethics committee approved the study, and all patients gave informed consent before enrollment.

### 2.2 Data acquisition

A number of 571 cases were selected; we excluded 146 patients, and 425 patients with cirrhosis were included in the study. The cirrhosis diagnosis was confirmed based on histopathological, clinical, and laboratory findings. Patients' data, including age, sex, comorbidities, the underlying cause of cirrhosis, complications of cirrhosis (i.e., ascites, portal hypertension, esophageal varices, and splenomegaly), and laboratory and ECG findings were recorded. The Child‐Pugh score and classification were calculated for included patients; class A: score of 5–6, class B: score of 7–9, and class C: score of 10–15.^5^

### 2.3 ECG analysis

A 12‐lead standard ECG with the calibrated device was performed for all patients on admission. Two experienced cardiologists, blinded to patients' information, reviewed the ECGs. P‐wave, PR interval, QRS complex, ST segment, T wave, and U wave were evaluated in all ECGs. QT intervals were calculated in the DII and V6 leads; the longest QT interval was considered and the average QT interval from three consecutive heartbeats was recorded. The corrected QT (QTc) interval was calculated by Bazett's formula (QTc = QT/√RR).^6^ Prolonged QTc was defined as >450 ms in men and >470 ms in women; patients were further divided into two groups of normal and prolonged QTc. R‐wave progression was categorized as normal (if transition zone appeared from the V3 or V4 leads) and poor (If S‐wave was dominant in all precordial leads).^7^

### 2.4 Statistical analysis

All statistical analyses were performed using the Statistical Package for the Social Sciences (SPSS Inc), Version 25. Normal distribution was assessed with Kolmogorov–Smirnov and Shapiro–Wilk tests. Continuous variables are reported as mean ± standard deviation or median (interquartile range), and categorical variables are presented as number (percentage). One‐way ANOVA/Kruskal–Wallis and *χ* ^2^ tests were used, and a two‐sided *p* < .05 was considered significant.

## 3 RESULTS

A total of 425 patients were included; the median (interquartile range) age was 36^8^ years, and 245 patients (57.6%) were men. The median serum potassium was 4.1 (0.4) mEq/L. Also, the prevalence of ascites was as follows: 265 (62.4%) with no ascites, 86 (20.2%) with mild ascites, 40 (9.4%) with moderate ascites, and 34 (8%) with severe ascites. Based on the ultrasounds of portal system and abdomen, 336 (79.1%) participants had portal hypertension, and 303 (71.3%) had splenomegaly. In 224 (52.7%) patients, the endoscopic investigation showed evidence of esophageal varices. Cryptogenic and primary sclerosing cholangitis (PSC) were the most common etiologies of cirrhosis. Moreover, 151 (35.5%) were in the Child‐Pugh score class A, 203 (47.8%) patients were in class B, and the rest were in class C. Prolonged QTc was present in 105 (24.7%) patients.

All ECG variables except for right ventricular hypertrophy (RVH) were related to the etiology of cirrhosis (Table 1). In those with early transitional zone and poor R wave progression (PRWP), PSC was the most common cause of cirrhosis. Cryptogenic cirrhosis was the most abundant in people with left ventricular hypertrophy (LVH), RVH, and prolonged QTc.

**Table 1:** The frequency of patients' characteristics in different liver cirrhosis etiologies.

Variables		Liver cirrhosis etiologies										*p* Value^a^
		BA *n* = 4	Cryptogenic *n* = 119	AIH *n* = 57	BCS *n* = 15	Hemangioma *n* = 18	PSC *n* = 116	ALD *n* = 15	VH *n* = 41	WD n = 27	NAFLD *n* = 13	
Ascites	Mild	‐	26 (30.2)	18 (20.9)	8 (9.3)	2 (2.3)	14 (16.3)	2 (2.3)	6 (7)	8 (9.3)	2 (2.3)	<.0001
	Moderate	‐	20 (50)	10 (25)	‐	‐	2 (5)	‐	4 (10)	4 (10)	‐	
	Severe	‐	6 (17.6)	6 (17.6)	2 (5.9)	‐	10 (29.4)	4 (11.8)	4 (11.8)	‐	2 (5.9)	
Portal HTN	Yes	4 (1.2)	94 (28)	53 (15.8)	15 (4.5)	9 (2.7)	76 (22.6)	15 (4.5)	34 (10.1)	23 (6.8)	13 (3.9)	<.0001
	No	‐	25 (28.1)	4 (4.5)	‐	9 (10.1)	40 (44.9)	‐	7 (7.9)	4 (4.5)	‐	
Esophageal varices	Yes	2 (0.9)	63 (28.1)	40 (17.9)	11 (4.9)	2 (0.9)	46 (20.5)	15 (6.7)	26 (11.6)	8 (3.6)	11 (4.9)	<.0001
	No	2 (1)	56 (27.9)	17 (8.5)	4 (2)	16 (8)	70 (34.8)	‐	15 (7.5)	19 (9.5)	2 (1)	
Splenomegaly	Yes	2 (0.7)	78 (25.7)	48 (15.8)	12 (4)	9 (3)	70 (23.1)	15 (5)	35 (11.6)	22 (7.3)	12 (4)	<.0001
	No	2 (1.6)	41 (33.6)	9 (7.4)	3 (2.5)	9 (7.4)	46 (37.7)	‐	6 (4.9)	5 (4.1)	1 (0.8)	
*Child‐Pugh class*												
*A*	2	48	6	4	16	50	4	10	11	‐		
*B*	2	51	30	11	2	64	4	18	10	11		<.0001
*C*	‐	20	21	‐	‐	2	7	13	6	2		
ETZ	Yes	‐	22 (26.2)	20 (23.8)	2 (2.4)	‐	24 (28.6)	2 (2.4)	10 (11.9)	‐	4 (4.8)	.006
	No	4 (1.2)	97 (28.4)	37 (10.9)	13 (3.8)	18 (5.3)	92 (27)	13 (3.8)	31 (9.1)	27 (7.9)	9 (2.6)	
PRWP	Yes	‐	4 (10)	4 (10)	6 (15)	4 (10)	12 (30)	2 (5)	2 (5)	2 (5)	4 (10)	<.0001
	No	4 (1)	115 (29.9)	53 (13.8)	9 (2.3)	14 (3.6)	104 (27)	13 (3.4)	39 (10.1)	25 (6.5)	9 (2.3)	
LVH	Yes	‐	20 (50)	8 (20)	‐	‐	2 (5)	‐	6 (15)	4 (10)	‐	.002
	No	4 (1)	99 (25.7)	49 (12.7)	15 (3.9)	18 (4.7)	114 (29.6)	15 (3.9)	35 (9.1)	23 (6)	13 (3.4)	
RVH	Yes	‐	6 (60)	2 (20)	‐	2 (20)	‐	‐	‐	‐	‐	.075
	No	4 (1)	113 (27.2)	55 (13.3)	15 (3.6)	16 (3.9)	116 (28)	15 (3.6)	41 (9.9)	27 (6.5)	13 (3.1)	
QTc	Prolonged	‐	24 (22.9)	21 (20)	4 (3.8)	2 (1.9)	22 (21)	8 (7.6)	10 (9.5)	8 (7.6)	6 (5.7)	.011
	Normal	4 (1.3)	93 (29.5)	36 (11.4)	11 (3.5)	16 (5.1)	92 (29.2)	7 (2.2)	31 (9.8)	19 (6)	6 (1.9)	

Abbreviations: AIH, autoimmune hepatitis; ALD, alcoholic liver disease; BA, biliary atresia; BCS, Budd‐Chiari syndrome; ETZ, early transitional zone; LVH, left ventricular hypertrophy; NAFLD, nonalcoholic fatty liver disease; portal HTN, portal hypertension; PRWP, poor r wave progression; PSC, primary sclerosing cholangitis; RVH, right ventricular hypertrophy; VH, viral hepatitis; WD, Wilson's disease.

^a^ *χ* ^2^ test.

QTc interval and early transitional zone were significantly related to the Child‐Pugh class (Table 2). Pairwise comparisons revealed that QTc in Child‐Pugh class A was significantly different from class B (*p* < .0001) and class C (*p* = .004), but QTc in class B and C were not statistically different.

**Table 2:** Demographic and ECG variables according to Child‐Pugh classification.

Variable		Total (*n* = 425)	Child‐Pugh class			*p* Value^a^
			A (*n* = 151)	B (*n* = 203)	C (*n* = 71)	
Age, *years*		36 (12)	35 (13)	36 (11)	37 (11)	.153
Gender, *male*		245 (57.6)	80 (52.9)	123 (60)	42 (59.1)	.340
QTc, *ms*		437 (46)	424 (53)	444 (46)	442 (35)	<.0001
Prolonged QTc	Yes	105 (24.7)	26 (17.4)	58 (29)	21 (29.6)	.03
	No	315 (74.1)	123 (82.6)	142 (71)	50 (70.4)	
Early transitional zone		84 (19.8)	16 (19)	52 (61.9)	16 (19)	.002
PRWP		40 (9.4)	20 (50)	14 (35)	6 (15)	.123
LVH		40 (10)	14 (35)	22 (55)	4 (10)	.433
RVH		10 (2.4)	6 (60)	2 (20)	2 (20)	.179

*Note*: Data are presented as median (interquartile range) or frequency (%).

Abbreviations: LVH, left ventricular hypertrophy; ms, milliseconds; PRWP, poor R wave progression; QTc, corrected QT interval; RVH, right ventricular hypertrophy.

^a^ Kruskal–Wallis and *χ* ^2^ tests.

## 4 DISCUSSION

**Figure 1:**
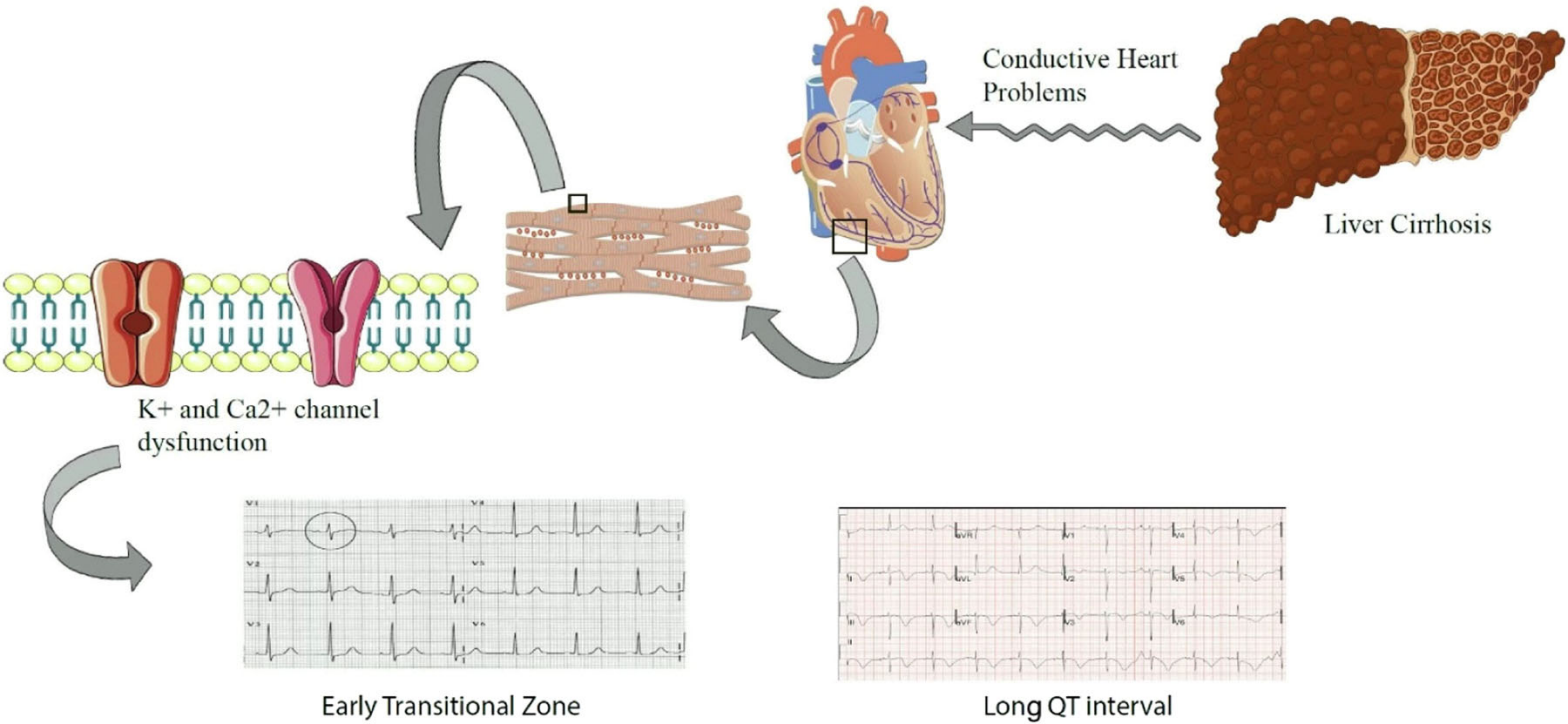
Effect of cirrhosis on ECG. Liver cirrhosis alters the function of membrane ion channels, such as Ca^2+^ and K^+^ channels. Decreased function of K^+^ channels as well as calcium channel dysfunction may explain the ECG changes observed in cirrhosis. ECG, electrocardiographic.

In this study, the most common etiologies for cirrhosis were cryptogenic and PSC. Early transitional zone, PRWP, LVH, and prolonged QT were significantly associated with cirrhosis etiology. Also, prolonged QT and early transitional zone were the most common ECG changes in this study, and were significantly related to the Child‐Pugh class (Figure 1).

Cardiac problems in patients with cirrhosis represent a clinical entity with significant effects on patient.^9^ A decrease in cardiac afterload causes cirrhotic patients to be asymptomatic at rest, but infection, hemorrhage, and exercise can lead to heart failure and serious cardiovascular complications.^8^ ECG can be beneficial in the early diagnosis of cardiovascular disorders in cirrhosis and reduce complications.^8^, ^9^, ^10^

In our study, QT prolongation was the most frequent ECG abnormality, similar to previous reports. Nevertheless, previous studies indicated that prolonged QT exists in about 50% of patients with cirrhosis,^11^, ^12^ which is higher than our findings. The difference may be attributed to different criteria; prolonged QT was formerly considered as >440 ms. Nonetheless, we used the up‐to‐date American Heart Association recommendation: QTc >450 ms for men and >470 ms for women.^13^ Our analyses also revealed that prolonged QT was significantly associated with Child‐Pugh class. There was no significant difference between class B and C. Still, QT was significantly lower in class A than in both classes B and C. The QT interval has been previously associated with poor prognosis among cirrhotic patients.^14^ In the cross‐sectional analysis of Lanzieri et al.^15^ patients in class C had longer QT intervals than class B and A. In a large cohort study, Ko et al.^16^ studied the preoperative, postoperative, and long‐term data of patients undergoing liver transplant. Prolonged QT was present in 65% of patients before liver transplant and significantly increased in the early postoperative period. But QT interval normalized in most of the patients after 6 months of the surgery, indicating that this feature of cirrhosis cardiomyopathy is reversible. The authors did not observe an association between prolonged QT and post‐op complications or mortality. Nevertheless, QT prolongation is reportedly associated with advanced disease in cirrhotic patients.^17^, ^18^ We also found that prolonged QT was significantly related to liver cirrhosis etiology and was most common in cryptogenic cirrhosis. This is in contrary to other findings showing that QT prolongation is more common in alcoholic liver disease^19^; our findings emphasize the incidence of this ECG change regardless of the etiology of cirrhosis. We recommend routine ECG examination to detect prolonged QT as a feature of cirrhotic cardiomyopathy; this can prevent serious adverse events such as sudden cardiac events in patients with cirrhosis.

Based on our findings, early transitional zone was the second most common ECG change in cirrhosis. It was also significantly related to cirrhosis etiology and Child‐Pugh class, indicating that this ECG change can determine the prognosis of affected individuals. In a normal ECG the size of the R wave is equal to or greater than the S wave, in V3 or V4 leads. There is an early transitional zone if this conversion occurs earlier (i.e., in V1 or V2). In general population, early transitional zone suggests increased cardiovascular risk and mortality.^20^ This ECG change may reflect the underlying hemodynamic changes associated with portal hypertension and cardiac dysfunction in cirrhotic individuals^21^; however, further investigations are warranted to confirm its efficacy in evaluating cardiac problems in cirrhosis.

As mentioned, cirrhotic cardiomyopathy produces a hyperdynamic state and impaired diastolic function caused by LVH. LVH was present in 40 (10%) of our study participants. According to Chen et al.,^22^ cirrhotic individuals have biventricular dilatation and dysfunction, but in our study, only 10 (2.4%) patients had ECG signs in favor of RVH. Neither LVH nor RVH was not significantly related to the Child‐Pugh class in our study, but there was a significant association between etiology of liver cirrhosis and LVH. None of our study participants had diseases affecting the ECG, including hypertension. Therefore, the presence of right or left ventricular hypertrophy in them is probably related to the complications of cirrhosis, especially portal hypertension and altered cardiac output.^23^ Other investigations using more advanced methods such as echocardiography and MRI, have found significant associations between chamber hypertrophy and severity of cirrhosis.^24^ Despite the valuable role of ECG in identifying heart disorders, we failed to find any association between ECG markers of LVH and RVH with Child‐Pugh score.

PRWP was present in 9.4% of our population and wasn't related to the Child‐Pugh class. To our knowledge, prior studies have scarcely investigated the significance of PRWP in patients with cirrhosis. This ECG change can be seen as a normal variant, or in anterior myocardial infarction, RVH, and LVH.^25^ Its presence in cirrhosis may indicate increased risk of heart failure.^26^ PRWP was not associated with the Child‐Pugh class in our study. Still, it has been known to predict sudden cardiac death and cardiovascular mortality in the general population.^27^, ^28^ Therefore, while PRWP on ECG is a nonspecific finding, it may have some clinical significance in cirrhotic patients and should prompt further evaluation for cardiac complications and careful monitoring for adverse outcomes.

The present study investigated the prevalence of several ECG changes in cirrhotic individuals and their association with Child‐Pugh score. However, the cross‐sectional study design is the major limitation of this study. Also, due to the heterogenicity of cirrhosis etiologies, larger sample sizes are needed to study the relationship between the etiology and ECG patterns. Further confirmatory studies are warranted to prove the relevance of using ECG changes as diagnostic methods for cardiac abnormalities and prognosis, in patients with cirrhosis.

## 5 CONCLUSION

ECG changes of prolonged QT and early transitional zone were related to the severity of cirrhosis. Using these signs may help in early diagnosis and management of cardiac dysfunction among cirrhotic patients leading to better prognosis.

## CONFLICT OF INTEREST STATEMENT

The authors declare no conflict of interest.

## ETHICS STATEMENT

The ethics committee of Shiraz University of Medical Sciences approved this study IR.SUMS.MED.REC.1399.508. All participants gave informed consent before enrollment.

## Data Availability

The data sets used or analyzed in the study are available upon reasonable request from the corresponding author.
